# Direct measurement of myocardial viability by manganese-enhanced MRI (MEMRI) tracks the regenerative effects by human pluripotent stem cell derived cardiomyocytes (hPCMs)

**DOI:** 10.1186/1532-429X-17-S1-P254

**Published:** 2015-02-03

**Authors:** Atsushi Tachibana, Eric Rulifson, Yuka Matsuura, Rahul Thakker, Maya Agarwal, Morteza Mahmoudi, Mouer Wang, Joseph C Wu, Rajesh Dash, Phillip Yang

**Affiliations:** Cardiovascular Medicine, Stanford University, Stanford, CA USA

## Background

Human pluripotent stem cell derived cardiomyocytes (hPCMs) may regenerate the myocardium to restore the cardiac function. Manganese-enhanced MRI (MEMRI) enters the cardiomyocytes via calcium channel to generate viability signal directly. Persistent engraftment of the hPCMs associated with increased myocardial viability and LVEF suggests regeneration. This study tests the hypothesis that hPCMs regenerate the injured murine myocardium.

## Methods

0.5 million human pluripotent stem cells and their cardiomyocyte derivatives were delivered into the injured SCID murine myocardium: iPSCs (n=3), hESCs (n=3), hCMs (n=3), iCMs (n=12), and PBS (n=4). MEMRI and cardiac MRI evaluated viability and function (3T HDx, GEHC, WI), using SeeMore IV (Eagle Vision, PA) on days 14 and 28 days post-MI. Bioluminescence imaging (BLI, Xenogen IVIS 200, MA) was performed to assess engraftment of the reporter gene transcued-iCMs (RG-iCMs).

## Results

The mean LVEF on days 14 and 28 post-MI demonstrated significant improvement in the hPCM groups vs. PBS control and undifferentiated cell groups. On days 14 and 28, the following LVEF measurements were observed: 1) Day 14 - iPSCs: 29.0±14.7%, hESCs: 25.8±9.2%, hCMs: 36.1±5.9%, iCMs: 33.1±4.4%, and PBS: 26.1±7.9% and 2) Day 28 - iPSCs: 20.6±7.6%, hESCs: 27.2±5.5%, hCMs: 44.1±9.5%*, iCMs: 35.1±8.4%*, and PBS: 18.9±3.8% (*p<0.05). Longitudinal LVEF change from days 14 to 28 demonstrated sustained functional improvement by hPCMs vs. PBS and undifferentiated cells: iPSCs: -8.4±8.2%, hESCs: 1.4±4.2%, hCMs: 7.9±3.9%*, iCMs: 2.0±4.0%*, and PBS: -7.2±4.9% (*p<0.05). Furthermore, hPCMs increased the % MEMRI measurement of myocardial viability (normalized to the total LV volume) from Day 14 to 28 - iPSCs: -6.6±5.8%, hESCs: -3.5±6.9%, hCMs: 6.1±5.5%*, iCMs: 2.3±0.3%* and PBS: -17.4%. Finally, the mean BLI signal, indicating iCM engraftment, demonstrated on days 14 (n=7) is 1.3±0.52 x10^3^p/s/cm^2^/sr and days 28 (n=3) is 4.2±2.2 x10^3^p/s/cm^2^/sr. Significant correlation was observed between LVEF and MEMRI in the hPCM groups (r=0.81, p=0.001) and between LVEF and BLI in the iCM group (r=0.66, p=0.04).

## Conclusions

Multimodality MRI-BLI platform allows reliable in vivo detection of myocardial regeneration through evaluation of stem cell engraftment, myocardial viability, and cardiac function. The findings suggest that the regenerative effects of hPCMs sustain the restoration of the injured myocardium.

## Funding

NIH/NHLBI UM1 (PY).

**Figure 1:**
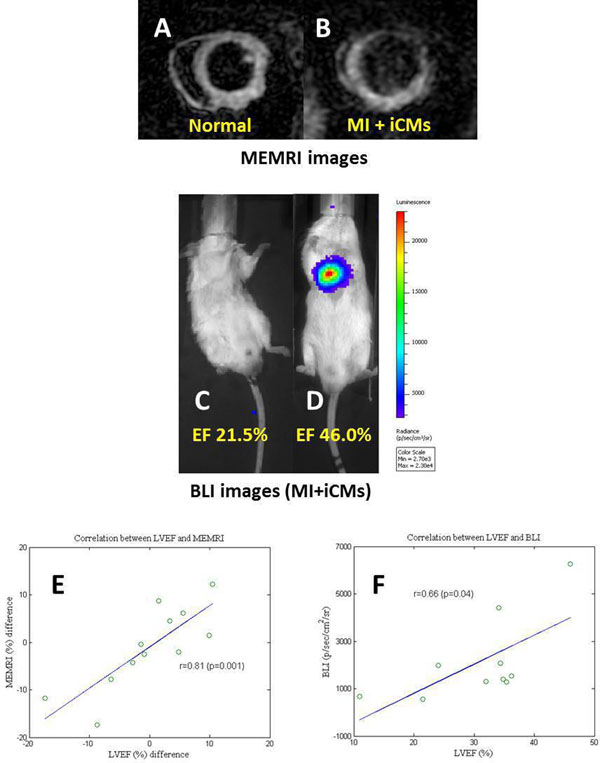
MEMRI images of healthy myocardium (A) and MI+iCMs (B). BLI signal was proportional to the LVEF (C,D), and good correlation (r=0.66, p=0.04) for MI+iCMs study (F). LVEF and MEMRI volume changes from week2 and week4 were significant correlation (r=0.81, p=0.001) between each cells (E).

